# Draft Genome Sequence of Loktanella maritima Strain YPC211, a Commensal Bacterium of the American Lobster (Homarus americanus)

**DOI:** 10.1128/genomeA.00314-18

**Published:** 2018-05-03

**Authors:** Hilary J. Ranson, Jason LaPorte, Edward Spinard, Marta Gomez-Chiarri, David R. Nelson, David C. Rowley

**Affiliations:** a Department of Biomedical and Pharmaceutical Sciences, University of Rhode Island, Kingston, Rhode Island, USA; b Department of Cell and Molecular Biology, University of Rhode Island, Kingston, Rhode Island, USA; c Department of Fisheries, Animal and Veterinary Sciences, University of Rhode Island, Kingston, Rhode Island, USA

## Abstract

Loktanella maritima strain YPC211 was isolated from the American lobster (Homarus americanus). We report here the draft genome sequence for L. maritima YPC211 and identify genes of potential importance to its role within the microbial community.

## GENOME ANNOUNCEMENT

Loktanella maritima is a Gram-negative aerobic bacterium that was originally isolated from shallow marine sediments in the Sea of Japan (1). Loktanella spp. appear to be ubiquitously distributed in the oceans and have been isolated from Antarctic to subtropical environments (2, 3). The genus belongs to the class Alphaproteobacteria, family Rhodobacterales, and order Rhodobacteraceae. Van Trappen et al. (2) originally proposed the genus to accommodate 3 species, but it has since been expanded and modified to contain 15 species (4–6). The strain Ha06YPC211 produces a beige/yellow pigment and was isolated from an egg mass of an American lobster (Homarus americanus) from Jamestown, RI.

L. maritima strain YPC211 was grown in artificial seawater (Instant Ocean) supplemented with yeast extract (1 g/liter) and peptone (5 g/liter) at 25°C on an elliptical shaker (New Brunswick) for 24 h. Genomic DNA was isolated using the Promega Wizard DNA purification kit, and DNA was resuspended in 2 mM Tris-HCl buffer (Bio Basic, Inc.). DNA was quantified using a NanoDrop 1000 spectrophotometer (ND-1000) and checked for quality on a 1% agarose gel stained with ethidium bromide. The DNA was sequenced on an Illumina MiSeq sequencer at the Genomics and Sequencing Center at University of Rhode Island. Reads were trimmed using CLC Genomic Workbench (version 8.5.1) for quality, ambiguous base pairs, adaptors, duplicates, and size, resulting in 4,491,164 paired-end reads. The draft genome was assembled using the *de novo* assembly algorithm of the CLC Genomic Workbench and SPAdes assembler (version 3.1.1). Contigs with a coverage of >76 reads were processed using the CLC Microbial Genome Finishing module. The completed draft genome is composed of 13 contigs, averaging 264,067 bp in size and 3 plasmids of 23,839, 6,277, and 215,371 bp in size (total genome, 3,678,360 bp), with an average G+C content of 53.5%. The draft genome was annotated using the Rapid Annotations using Subsystems Technology (RAST) server and resulted in 3,681 open reading frames (7).

The genome of L. maritima YPC211 encodes type I, II, III, and IV secretion systems. It has been reported that Loktanella spp. can survive temperature extremes and have been isolated from Antarctic mats (2). Accordingly, five putative cold shock proteins (including CspA, CspB, and CspC) were annotated. Additionally, the genome encodes four hemolysins, two metalloproteases, and one extracellular protease. Two iron acquisition systems, TonB and hemin (HmuS), were annotated along with iron-specific hemin and ABC transporters. Ferric siderophore-related genes were also identified. The RAST-annotated draft genome was submitted to Antibiotics and Secondary Metabolite Analysis Shell (antiSMASH) for secondary metabolite biosynthesis gene cluster analysis (8), leading to the identification of four clusters, including a nonribosomal peptide synthetase, type I polyketide synthase, bacteriocin, and homoserine lactone biosynthetic gene clusters. L. maritima YPC211 inhibits the growth of the shrimp pathogen Vibrio parahaemolyticus when cocultivated on an agar surface, as determined by a zone of inhibition assay (9). Further investigation of these biosynthetic gene clusters may aid in characterization of compounds responsible for this antibiosis.

### Accession number(s).

This whole-genome shotgun project has been deposited in DDBJ/ENA/GenBank under the accession number PKFN00000000. The version described in this paper is the first version, PKFN01000000.
